# The Inhibition of microRNA-128 on IGF-1-Activating mTOR Signaling Involves in Temozolomide-Induced Glioma Cell Apoptotic Death

**DOI:** 10.1371/journal.pone.0167096

**Published:** 2016-11-28

**Authors:** Peng-Hsu Chen, Chia-Hsiung Cheng, Chwen-Ming Shih, Kuo-Hao Ho, Cheng-Wei Lin, Chin-Cheng Lee, Ann-Jeng Liu, Cheng-Kuei Chang, Ku-Chung Chen

**Affiliations:** 1 Graduate Institute of Medical Sciences, College of Medicine, Taipei Medical University, Taipei, Taiwan; 2 Department of Biochemistry and Molecular Cell Biology, School of Medicine, College of Medicine, Taipei Medical University, Taipei, Taiwan; 3 Department of Clinical Pharmacy, School of Pharmacy, Taipei Medical University, Taipei, Taiwan; 4 Department of Pathology and Laboratory Medicine, Shin Kong Wu Ho-Su Memorial Hospital, Taipei, Taiwan; 5 Department of Neurosurgery, Taipei City Hospital Ren-Ai Branch, Taipei, Taiwan; 6 Department of Neurosurgery, Shuang Ho Hospital, Taipei Medical University, New Taipei City, Taiwan; 7 Graduate Institute of Injury Prevention and Control, Taipei Medical University, Taipei, Taiwan; Thomas Jefferson University, UNITED STATES

## Abstract

Temozolomide (TMZ), an alkylating agent of the imidazotetrazine series, is a first-line chemotherapeutic drug used in the clinical therapy of glioblastoma multiforme, the most common and high-grade primary glioma in adults. Micro (mi)RNAs, which are small noncoding RNAs, post-transcriptionally regulate gene expressions and are involved in gliomagenesis. However, no studies have reported relationships between TMZ and miRNA gene regulation. We investigated TMZ-mediated miRNA profiles and its molecular mechanisms underlying the induction of glioma cell death. By performing miRNA microarray and bioinformatics analyses, we observed that expression of 248 miRNAs was altered, including five significantly upregulated and 17 significantly downregulated miRNAs, in TMZ-treated U87MG cells. miR-128 expression levels were lower in different glioma cells and strongly associated with poor survival. TMZ treatment significantly upregulated miR-128 expression. TMZ significantly enhanced miR-128-1 promoter activity and transcriptionally regulated miR-128 levels through c-Jun N-terminal kinase 2/c-Jun pathways. The overexpression and knockdown of miR-128 expression significantly affected TMZ-mediated cell viability and apoptosis-related protein expression. Furthermore, the overexpression of miR-128 alone enhanced apoptotic death of glioma cells through caspase-3/9 activation, poly(ADP ribose) polymerase degradation, reactive oxygen species generation, mitochondrial membrane potential loss, and non-protective autophagy formation. Finally, we identified that key members in mammalian target of rapamycin (mTOR) signaling including mTOR, rapamycin-insensitive companion of mTOR, insulin-like growth factor 1, and PIK3R1, but not PDK1, were direct target genes of miR-128. TMZ inhibited mTOR signaling through miR-128 regulation. These results indicate that miR-128-inhibited mTOR signaling is involved in TMZ-mediated cytotoxicity. Our findings may provide a better understanding of cytotoxic mechanisms of TMZ involved in glioblastoma development.

## Introduction

Glioblastoma multiforme (GBM), a grade IV histological malignancy according to the World Health Organization (WHO) classification, is the most common and aggressive primary brain tumor with a poor prognosis in adults [1, 2]. More than 90% of patients with GBM have primary gliomas. The average survival duration of these patients is less than 6 months. Malignant gliomas are highly mobile, invasive, and difficult to completely resect through surgery [3]. Therefore, radiation and chemotherapy are generally performed as adjuvant therapies after surgical treatment. Temozolomide (TMZ), which can penetrate the blood–brain barrier, is an alkylating agent of the imidazotetrazine series and a major chemotherapeutic drug for clinical treatment of malignant gliomas [4]. Because of the malignant progression and widespread invasion of GBM throughout the brain, the gradually increasing drug resistance of TMZ reduces its therapeutic effects on patients. In addition, the course of TMZ treatment is administered until patients’ death and thus may cause financial burdens. Therefore, elucidation of TMZ-mediated gene networks may facilitate the development of innovative therapeutic strategies for drug designs and clinical therapies for glioblastomas.

Micro (mi)RNAs are novel class of endogenous, small, noncoding RNAs that control gene expression by binding to their target messenger (m)RNAs for degradation and translational repression. Aberrant miRNA levels regulate various cellular processes such as differentiation, proliferation, and apoptosis. Several miRNAs involved in GBM development have been recently identified [5]. For example, miR-10b, miR-17-92 cluster, miR-21, and miR-93 are overexpressed in GBM [6]. Compared with normal brain tissues, miR-7, miR-34a, miR-128, and miR-137 are significantly downregulated in gliomas. Furthermore, miR-128, a brain-enriched miRNA, plays a critical role in regulating the development of the nervous system and its normal physiological functions [7]. Aberrant miR-128 expressions have been reported in many malignant tumor types, particularly in GBM [8]. miR-128 is an intronic miRNA encoded by two distinct genes, miR-128-1 and miR-128-2, in introns of R3H domain containing 1 (*R3HDM1*) and cyclic AMP-regulated phosphoprotein, 21 kDa (*ARPP-21*) genes, respectively. Furthermore, miR-128 can induce glioma cell death and inhibit migration through targeting RhoE [9], EphB2 [10], p70S6K1 [11], Bmi-1 [12], and E2F3a [13]. Serum miR-128 levels can be used as a sensitive and specific biomarker of gliomas [14]. Although increasing evidence has suggested the functions of miR-128, molecular mechanisms underlying the transcriptional regulation of *miR-128* genes in gliomas remain unclear.

Mammalian target of rapamycin (mTOR), a member of the serine/threonine protein kinase family, forms protein complexes that regulate cell growth and proliferation [15]. mTOR binds to rapamycin-insensitive companion of mTOR (RICTOR) and regulatory-associated protein of mTOR (RAPTOR) and forms two functionally distinct complexes in mammalian cells, mTOR complex 1 (mTORC1) and mTORC2. The functions of mTORC1 are well-established, and coordinate cell growth and promotion. However, functions of mTORC2 are not completely clear. The upstream regulators of the canonical mTOR signal cascade and its downstream targets, p70S6K1 and 4E-BP1, are induced by growth factors such as insulin-like growth factor (IGF)-1 through PI3K/PDK1/AKT pathways, [16]. In the brain, mTORC1 controls protein translation to regulate synaptic plasticity, memory, learning, and GBM pathogenesis [17]. Several studies have suggested that abnormal mTOR signaling is a crucial therapeutic target for GBM. Therefore, elucidation of mechanisms underlying the inhibition of mTOR signaling can provide significant and novel directions for clinical treatment of GBM.

Although TMZ is a first-line chemotherapeutic drug for GBM, no studies have reported the effects of TMZ on miRNA gene regulation. In this study, we conducted a miRNA microarray analysis of TMZ-treated U87MG cells to identify TMZ-mediated miRNA profiles. In addition, we validated the upregulation of miR-128 expression by TMZ in glioma cell apoptotic death through c-Jun N-terminal kinase 2 (JNK2)/c-Jun pathways. Finally, we investigated the mTOR signaling pathways inhibited by miR-128 in TMZ cytotoxicity.

## Materials and Methods

### Chemicals and reagents

Human glioblastoma Hs683, M059K, and U87MG cells were purchased from the Bioresource Collection and Research Center (Hsinchu City, Taiwan). Primary human astrocytes were purchased from Thermo Fisher Scientific (Waltham, MA, USA). Other cell culture-related reagents were purchased from Gibco-BRL (Grand Island, NY, USA). Anticaspase-3, JNK, c-Jun, phosphorylated (p)-JNK, and p-c-Jun antibodies were purchased from Cell Signaling Technology (Danvers, MA, USA). All other antibodies were purchased from GeneTex (Hsinchu City, Taiwan). TMZ, 3-methyladenine (3-MA), H_2_DCFHDA, rhodamine 123, acridine orange, annexin V, propidium iodide (PI), 3-(4,5-dimethylthiazol-2-yl)-2, 5-diphenyl tetrazolium bromide (MTT), U0126, SB203580 (SB), and SP600125 (SP) were purchased from Sigma-Aldrich (St. Louis, MO, USA). EZ-ChIP™, polyvinylidene difluoride (PVDF) membranes, and enhanced chemiluminescence (ECL) solution were purchased from Millipore (Billerica, MA, USA). Trizol® reagent, Lipofectamine 3000, and secondary antibodies were purchased from Invitrogen (Thermo Fisher Scientific). SYBR® Green PCR master mix; MultiScribe (tm) reverse transcriptase kit; and TaqMan® pri-miR-128-1, pri-miR-128-2, miR-128, and U6B assays were purchased from Applied Biosystems (Thermo Fisher Scientific). The dual luciferase reporter assay and Nano-Glo dual luciferase assay systems were purchased from Promega (Madison, WI, USA). The miR-128 inhibitor was purchased from GenePharma **(**Suzhou, China). Primer sets were synthesized by Genomics BioSci & Tech (Xizhi, New Taipei City, Taiwan). All short hairpin (sh)RNAs were purchased from the National RNAi Core Facility (Nankang, Taiwan). Unless otherwise specified, all other reagents were of analytical grade.

### Cell culture, treatments, and transfection

U87MG cells were maintained in minimum essential Eagle’s medium; M059K cells were maintained in a 1:1 mixture of Dulbecco’s modified Eagle’s medium (DMEM) and Ham’s F12 medium containing 2.5 mM L-glutamine; HS683 cells were maintained in DMEM containing 4 mM L-glutamine; and astrocytes were maintained in DMEM containing N-2 supplement. All cells were supplemented with 10% fetal bovine serum (Biological Industries, Cromwell, CT, USA), 100 units/mL penicillin, 100 μg/mL streptomycin, 1 mM sodium pyruvate, and 1 mM nonessential amino acids at 37°C in a 5% CO_2_ incubator. For TMZ treatment, different or indicated doses of TMZ were added to overnight-cultured cells for the indicated times. For mitogen-activated protein kinase (MAPK) inhibitor treatment, 10 μM U0126, 5 μM SB203580, and 10 μM SP600125 were respectively pretreated for 1 h and TMZ was then added to overnight-cultured cells for another 24 h. For transfection experiments, cells were seeded into a 12-well plate at a density of 10^5^ cells/well. After achieving 70% confluence in a well, indicated doses of miR-128 plasmids, 100 nM of the miR-128 inhibitor, scrambled/gene shRNAs (1 μg), and 500 ng of the pGL3-promoter reporter plasmids were respectively transfected with Lipofectamine 3000 (Invitrogen) according to the manufacturer’s instructions. After 24 h of incubation, cells were lysed for further experiments.

### Cell viability assay

Cell viability was assessed using the MTT assay. Cells were seeded on a 96-well plate at a density of 8 × 10^3^ cells/well overnight, followed by treatment with various TMZ concentrations for another 24 h. Before the end of treatment, 0.5 mg/mL MTT was added to each well for 4 h. Supernatants were carefully aspirated, and formazan crystals were dissolved using dimethyl sulfoxide (DMSO). The absorbance was measured at 550 nm by using the Thermo Varioskan Flash reader (Thermo Fisher Scientific, Waltham, MA, USA).

### Immunoblot analysis

Cells were harvested in RIPA buffer [1% Nonidet P-40, 0.5% deoxycholate, and 0.1% sodium dodecylsulfate (SDS) in phosphate-buffered saline (PBS)] containing a protease inhibitor cocktail (Calbiochem, Billerica, MA, USA) and centrifuged at 12,000 rpm for 10 min at 4°C. The supernatant was used as the total cell lysate. Lysates (20 μg) were denatured in 2% SDS, 10 mM dithiothreitol, 60 mM Tris-hydrochloric acid (Tris-HCl, pH 6.8), and 0.1% bromophenol blue and loaded onto a 10%–15% polyacrylamide/SDS gel. Separated proteins were then transferred to a PVDF membrane. The membrane was blocked for 1 h at room temperature in PBS containing 5% nonfat dry milk and incubated overnight at 4°C in PBS-T containing the primary antibody. The membrane was washed in PBS-T, incubated with the secondary antibody conjugated to horseradish peroxidase for 1 h at room temperature, and then washed in PBS-T. An ECL nonradioactive detection system was used to detect antibody–protein complexes.

### Microarray, pathway analysis, and survival rate analysis

Total RNA was respectively isolated from U87MG cells with or without 400 μM TMZ treatment for 24 h. miRNA expression profiling was performed using Human microRNA OneArray^®^, Version 6.2 (Phalanx Biotech Group, Hsinchu, Taiwan). All experiments including complementary (c)RNA amplification, hybridization, image scanning with an Axon 4000 scanner (Molecular Devices, Sunnyvale, CA, USA), and statistical analysis using the Genepix software (Molecular Devices) were conducted by the Phalanx Biotech Group (Hsinchu, Taiwan). The log2 (ratio) was calculated by the pairwise combination and error-weighted average. Significantly differentially expressed (DE) gene lists filtered by a *p* value of less than 0.05 and a |log2 (ratio)| of more than or equal to 0.58 or less than or equal to −0.51 cutoff were applied for further analysis. Hierarchical clustering analysis and heatmap visualization were performed using CIMminer [18]. Target genes of miRNAs were predicted using TargetScan 6.2 [19]. For the survival rate analysis, TCGA array data were analyzed using the SurvExpress platform [20]. All probe sets were averaged per sample. Statistical analysis was performed using the Kaplan–Meier log-rank test and Cox proportional hazard regression to determine the relationship between gene expression and survival time. High- and low-risk groups were categorized according to significantly different survival rates.

### RNA isolation and real-time reverse transcription-quantitative polymerase chain reaction

Total RNA from cultured cells was extracted using the Trizol® reagent according to manufacturer’s instructions. RNA quality was examined using A260/A280 readings. cDNA was synthesized from 1 μg of total RNA by using a random primer and the MultiScribe (tm) reverse transcriptase kit. cDNA was diluted at a ratio of 1:30 with polymerase chain reaction (PCR)-grade water and then stored at −20°C. To detect miRNA and U6B, cDNA was synthesized using TaqMan® microRNA Assays. The kits (Applied Biosystems) used could specifically and separately detect miR-128, pri-miR-128-1, and pri-miR-128-2. Specific primers for human genes and 18s ribosomal (r)RNA used in real-time quantitative PCR (qPCR) are listed in S1 Table. Gene expression levels were quantified using the Applied Biosystem StepOnePlus™ system (Thermo Fisher Scientific) under preoptimized conditions. Each PCR was performed in triplicate by using 5 μL 2× SYBR Green PCR master mix, 0.2 μL of primer sets, 1 μL cDNA, and 3.6 μL nucleotide-free H_2_O to obtain a yield of 10 μL per reaction. Expression rates were calculated as the normalized C_T_ difference between the control and sample after adjusting for amplification efficiency relative to the expression level of the housekeeping gene 18s rRNA. To quantify miRNA expression levels, U6B was used as an internal control.

### Construction of the *miR-128* promoter reporter plasmid and mutagenesis

To investigate the effect of TMZ on the activity of *miR-128* promoters, several reporter constructs were produced. A 2.5-kb fragment of *miR-128-1* and *miR-128-2* promoters was respectively isolated through PCR by using primers listed in S1 Table. After digestion of the PCR product with *Mlu*I and *Hind*III, the insert was cloned into the pGL3 reporter vector (Promega), creating the expression vectors pGL3-miR-128-1-prom and pGL3-128-2-prom. After sequencing, pGL3-miR-128-1-prom was used as a template, and pGL3-miR-128-2K, pGL3-miR-128-1.5K, pGL3-miR-128-1K, and pGL3-miR-128-0.5K were respectively isolated through PCR. Site-directed mutagenesis of c-Jun-binding sites in *miR-128* promoters was performed using the QuikChange® site-directed mutagenesis kit (Stratagene, Heidelberg, Germany) and named pGL3-miR-128-1-prom-MUT, for which pGL3-miR-128-1-prom was used as a template. For reporter assays, cells were transiently transfected with different sizes of wild-type or mutant reporter plasmids and then treated with TMZ using Lipofectamine 3000 (Invitrogen). pNL1.1-TK plasmids were cotransfected and served as an internal control. The reporter assay was performed 24 h after transfection by using the Nano-Glo dual luciferase assay system (Promega).

### Chromatin immunoprecipitation assay

Chromatin immunoprecipitation (ChIP) assays were performed according to manufacturer’s instructions (EZ-ChIP^TM^, Millipore). Briefly, 10^6^ cells with or without TMZ treatment were fixed with 1% formaldehyde, washed with cold PBS, and lysed in a buffer. Nuclei were sonicated to shear the DNA, and lysates were pelleted and precleared. Protein–DNA complexes were incubated with 1 μg of the c-Jun antibody overnight and then incubated with protein G beads, followed by elution in 1% SDS/0.1 M NaHCO_3_ and reverse crosslinking at 65°C. DNA recovered from samples containing the c-Jun antibody was compared with a negative control [mouse immunoglobulin G (IgG)] and positive control (an anti-RNA-Pol antibody) provided by the manufacturer. Finally, DNA was subjected to PCR analysis after recovery. PCR primers are listed in S1 Table.

### Construction of miR-128-overexpressing plasmids

A 300-bp length of the miR-128 gene was generated through PCR amplification by using primers listed in S1 Table. The following thermal profile was used for PCR amplification of 500 ng of genomic DNA on a GeneAmp PCR system 9700 (Applied Biosystems): an initial denaturation step at 95°C for 5 min, followed by 40 cycles of 94°C for 1 min, 58°C for 1 min, and 72°C for 1 min, with a final extension at 72°C for 10 min. PCR products were analyzed through agarose gel electrophoresis. All PCR products were cloned into the pCDH vector (System Biosciences, Palo Alto, CA, USA) and sequenced. After *Bam*HI/*Eco*RI digestion, the *miR-128* gene was cloned into pCDH to form a construct named pCDH-miR-128.

### Detection of apoptosis

Apoptosis was analyzed through flow cytometry with annexin V/PI double staining to detect membrane events. In brief, after transfection with the indicated dose of pCDH-miR-128 for 24 h, whole cells were collected in Hepes buffer containing 10 mM Hepes (pH 7.4), 140 mM NaCl, and 2.5 mM CaCl_2_. Subsequently, cells were stained with annexin V (2.5 μg/mL) and PI (2 ng/mL) for 20 min, followed by analysis on a flow cytometer using the CellQuest software (Becton Dickinson, San Jose, CA, USA). The four quadrants of the cytogram in figures were used to distinguish normal (annexin V^−^/PI^−^), early apoptotic (annexin V^+^/PI^−^), late apoptotic (annexin V^+^/PI^+^), and necrotic (annexin V^−^/PI^+^) cells. The sum of early apoptosis and late apoptosis was presented as total apoptosis.

### Measurement of reactive oxygen species

Intracellular levels of hydrogen peroxide (H_2_O_2_) were detected using the H_2_DCFHDA stain. H_2_O_2_ oxidizes H_2_DCFHDA and then emits green fluorescence. Cells were collected after transfection with indicated doses of pCDH-miR-128 for 24 h. Then, cells were resuspended and stained with 10 μM H_2_DCFH-DA for 30 min at 37°C in the dark. Fluorescence was measured on a flow cytometer by using the CellQuest software. The percentage increase in the fluorescence peak was used to represent the level of reactive oxygen species (ROS) production.

### Measurement of the mitochondrial membrane potential

The mitochondrial membrane potential (MMP) was measured through flow cytometry by using rhodamine-123, a cationic lipophilic fluorochrome. After transfection with the indicated dose of pCDH-miR-128 for 24 h, cells were again incubated with 20 nM rhodamine-123 at 37°C for 20 min. Then, cells were washed twice with PBS, and the intensity of rhodamine-123 was determined through flow cytometry. Fluorescence was measured using a flow cytometer and the CellQuest software. Cells with decreased fluorescence (less rhodamine-123) were counted as those cells that lost some of their MMP.

### Detection of acidic vesicular organelles

Autophagy is characterized by the formation and promotion of acidic vesicular organelles, which can be detected through acridine orange (AO) staining and flow cytometry. After cells were stained with AO, the cytoplasm and nuclei exhibited bright-green and dim-red fluorescence, respectively, whereas the acidic compartment exhibited bright-red fluorescence. Therefore, the intensity change in red fluorescence could be represented as the percentage of the cellular acidic compartment. After transfection with the indicated dose of pCDH-miR-128 for 24 h, green (FL-1) and red (FL-3) fluorescence changes of AO-stained cells were measured using a flow cytometer and the CellQuest software. The sum of the upper-left and upper-right quadrants of the cytogram was used to represent the percentage autophagy.

### Construction of 3ʹ-untranslated region (UTR) reporter plasmid and mutagenesis

PCR was performed using primer sets specific to the mTOR/PDK1/RICTOR/IGF1/PIK3R1 3’-UTR, of which the forward and reverse primers were linked to *Xho*I and *Xba*I sites, respectively. PCR primers are listed in S1 Table. U87MG genomic DNA was used as a template. The PCR product was digested with *Xho*I and *Xba*I and cloned downstream of the luciferase gene in the pMIRGLO-REPORT luciferase vector (Promega). Site-directed mutagenesis of the miR-128 target site in the 3’-UTR was performed using the QuikChange site-directed mutagenesis kit (Stratagene). For reporter assays, cells were transiently transfected with wild-type or mutant reporter plasmids and miR-128-expressing plasmids by using Lipofectamine 3000 (Invitrogen). The reporter assay was performed 24 h after transfection by using the dual luciferase assay system (Promega). The dual Renilla luciferase value was used as an internal control.

### Statistical analysis

All data are presented as the mean ± standard deviation (SD). Significant differences among groups were determined using unpaired Student’s *t*-test. A value of *p* < 0.05 was considered as an indication of statistical significance. All figures presented were obtained from at least three independent experiments with similar results.

## Results

### Investigation of TMZ-mediated miRNA profiles

**Fig 1:**
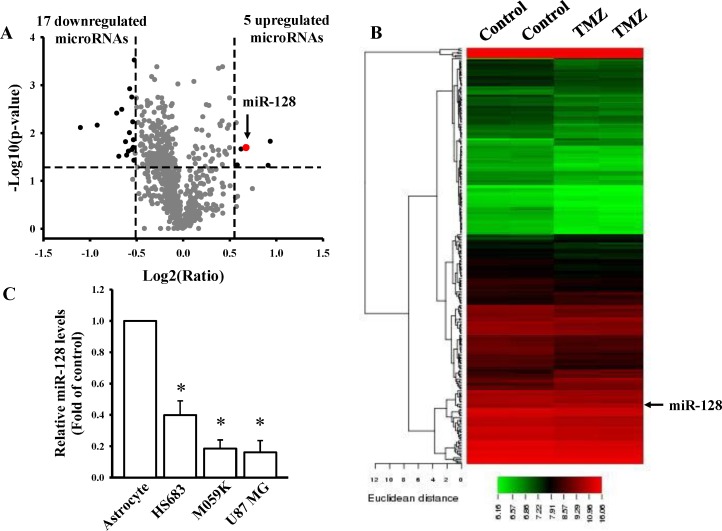
Investigation of TMZ-mediated microRNA profiles. Volcano plot (A) and heatmap of hierarchical gene clustering (B) demonstrating TMZ-regulated microRNA signatures. After U87MG cells were treated with 400 μM TMZ for 24 h, total RNAs were extracted for the microarray. For all array analyses, a *p* value of less than 0.05 and a log2 (ratio) of more than or equal to 0.58 or less than or equal to −0.51-multiple of change cutoff were applied. Volcano plots exhibit multiples of change (log_2_ ratio) and probabilities (-log_10_ *p* values) of individual microRNAs from the microarray assay. Significantly upregulated and downregulated mRNAs are indicated by black dots. The red dot indicates miR-128. The heatmap depicts the 248 differentially expressed microRNAs between TMZ subsets. A color was assigned to each microRNA based on its relative expression across samples. (C) Detection of endogenous miR-128 levels in normal human astrocytes and three different glioma cell lines including HS683, M059K, and U87MG cells. Relative expression levels of miR-128 were measured through real-time PCR. The U6B level was used as an internal control. Data are the mean ± SD of three experiments.

To examine the cytotoxic effects of TMZ on the viability of U87MG cells, cells were treated with different TMZ doses for 24 h (S1 Fig). We observed that TMZ induced U87MG cell death in a dose-dependent manner. Treatment with 400 μM TMZ for 24 h reduced the cell viability of U87MG cells by 62%. Then, we used this condition for conducting the miRNA microarray analysis. We selected significant DE miRNAs with a log2 (ratio) of more than or equal to 0.58 or less than or equal to −0.51 and a *p* value (differentially expressed) of less than 0.05 (Fig 1A). Among 248 DE miRNAs, TMZ treatment significantly upregulated the expression of five miRNAs and downregulated the expression of 17 miRNAs (Fig 1B, Table 1, and S1 File). Because miR-128 has been reported to have a tumor suppressor role in inhibiting gliomagenesis [21], we next investigated the effects of TMZ on the regulation of miR-128 genes.

**Table 1:** List of temozolomide (TMZ)-mediated microRNAs.

microRNA	Normalized Intensity		Log2 (Ratio)	*p* value
	Control	TMZ		
**TMZ-upregulated microRNAs [log2 (ratio) >0.58; *p* value <0.05]**				
hsa-miR-4634	87.68	167.99	0.94	0.02
hsa-miR-3178	407.94	769.41	0.92	0.05
hsa-miR-128	370.11	588.02	0.67	0.02
hsa-miR-320b	309.93	477.79	0.62	0.02
hsa-miR-22-3p	316.02	474.01	0.58	0.05
**TMZ-downregulated microRNAs [log2 (ratio) <-0.51; *p* value <0.05)**				
hsa-miR-4525	2129.75	993.79	-1.10	0.01
hsa-miR-4653-3p	5777.87	3055.70	-0.92	0.01
hsa-miR-765	5975.21	3652.64	-0.71	<0.01
hsa-miR-4505	5719.33	3552.36	-0.69	0.03
hsa-miR-6088	6953.73	4412.38	-0.66	<0.01
hsa-miR-4508	3423.75	2235.56	-0.61	0.02
hsa-miR-4271	1042.62	686.12	-0.60	0.03
hsa-miR-4689	2440.73	1627.54	-0.58	0.02
hsa-miR-4665-5p	465.15	312.63	-0.57	0.01
hsa-miR-4433-3p	416.68	280.54	-0.57	<0.01
hsa-miR-1225-5p	694.82	475.19	-0.55	<0.01
hsa-miR-1587	2541.92	1740.08	-0.55	0.02
hsa-miR-762	3498.19	2410.16	-0.54	0.01
hsa-miR-30c-1-3p	1172.16	810.70	-0.53	0.02
hsa-miR-21-3p	172.18	119.15	-0.53	0.01
hsa-miR-6076	625.15	434.37	-0.53	0.04
hsa-miR-4507	2404.18	1674.73	-0.52	<0.01

### Association of aberrant miR-128 levels with poor survival

**Fig 2:**
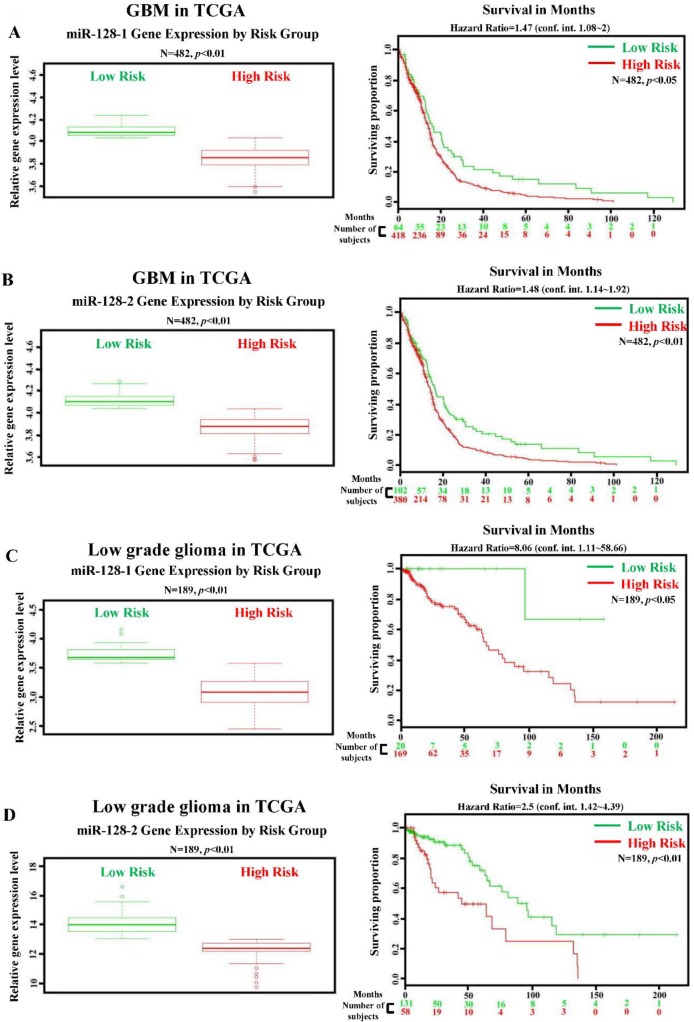
Lower miR-128 levels are significantly associated with a high-risk group and poor survival in patients with GBM and low-grade glioma. Correlation between miR-128-1/miR-128-2 levels and survival rates in patients with GBM (A and B) and low-grade glioma (C and D). The left panel shows relative miR-128-1/miR-128-2 expression levels according to the patient risk group based on GBM and low-grade glioma profiling from the TCGA database. High-risk individuals had lower miR-128-1/miR-128-2 expression levels, which are denoted by red symbols. By contrast, higher miR-128-1/miR-128-2 expression levels were associated with higher survival and are shown in green. The right panel shows results of the Kaplan–Meier analysis of survival data of patients with GBM and low-grade glioma profiles from the TCGA database. X axis values indicate the number of patients per group. Red curves indicate patients at a high risk. Green curves indicate patients at a low risk. The plus sign (+) indicates censored observations. *p* values were calculated using the log-rank test.

To explore the role of miR-128 in gliomagenesis, we first compared endogenous levels of miR-128 among four cell lines, namely primary astrocytes and HS683, M059K, and U87MG glioma cells. The expression level of miR-128 was lower than that of primary astrocytes in glioma cells (Fig 1C). The survival rate analysis performed using the TCGA database demonstrated a significant association of low expression levels of miR-128 including miR-128-1 and miR-128-2 with a high risk in and poor survival rate of patients with glioma (Fig 2A and 2B). Moreover, decreased miR-128 gene levels were significantly correlated with poor survival in patients with low-grade glioma (Fig 2C and 2D), suggesting that miR-128 plays a crucial role in the inhibition of gliomagenesis.

### TMZ transcriptionally upregulated the expression of *miR-128-1* but not of *miR-128-2*

**Fig 3:**
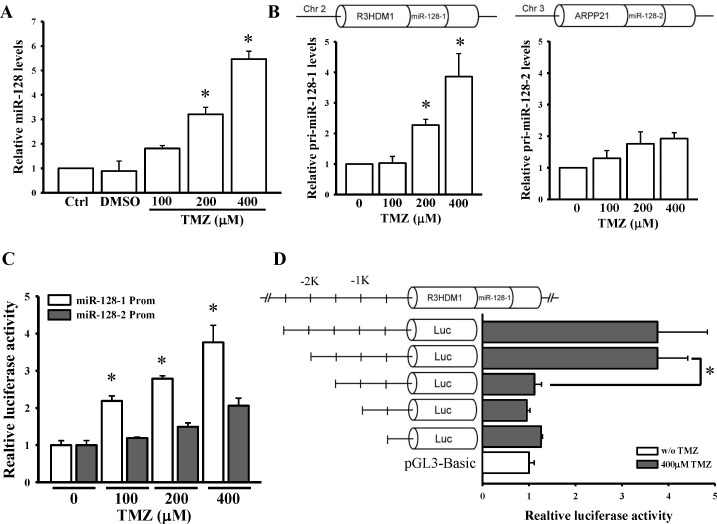
TMZ upregulates *miR-128-1* but not *miR-128-2* activation. (A) Dose-dependent effects of TMZ on mature (A) and primary (B) miR-128 expression levels. After cells were treated with the indicated doses of TMZ for 24 h, endogenous miR-128, primary (pri)-miR-128-1, and pri-miR-128-2 levels were respectively measured through real-time PCR. U6B and 18S rRNA levels were used as internal controls for the mature and primary miR-128 genes, respectively. (C) TMZ dose-dependent activated *miR-128-1* but not *miR-128-2* promoter activity. (D) Identification of the core region in the *miR-128-1* promoter. After cells were transfected with 500 ng pGL3-Basic, pGL3-miR-128-1-prom2500, or pGL3-128-1 variants for 24 h, luciferase activity was measured with TMZ treatment for another 24 h. pNL1.1.TK[*Nluc*/TK] plasmids at 5 ng were also cotransfected into cells, and the NanoLucR luciferase value was used as an internal control. Data are the mean ± SD of three experiments. * *p* < 0.05.

To reconfirm the microarray results, we evaluated the effects of TMZ on the expression levels of miR-128. As presented in Fig 3A, TMZ significantly upregulated the expression levels of miR-128 in a dose-dependent manner. miR-128 is an intronic miRNA and present in two gene loci, miR-128-1 in *R3HDM1* and miR-128-2 in *ARPP21* (Fig 3B). To investigate which of these gene loci are mainly regulated by TMZ, we first detected expression changes of two primary (pri) forms of miR-128 after TMZ treatment. As presented in Fig 3B, TMZ significantly upregulated the pri-miR-128-1 expression level and exerted a mild effect on pri-miR-128-2 expression. In addition, we compared endogenous levels of the two pri-miR-128 genes. No significant difference was observed between pri-miR-128-1 and pri-miR-128-2 levels (S2A Fig). Furthermore, the promoter reporter assay indicated that TMZ promoted only the gene activity of miR-128-1 in a dose-dependent manner but not that of *miR-128-2* (Fig 3C). Without TMZ treatment, the endogenous promoter activities of the two *miR-128* genes were similar (S2B Fig). To identify the core region determining miR-128-1 promoter activity, various fragments of the promoter were cloned. As depicted in Fig 3D, deletion of the region between −2000 and −1500 bp resulted in the most significant change in luciferase activity. Taken together, TMZ enhanced miR-128 expression through transcriptionally regulating the *miR-128-1* promoter.

### The JNK2/c-Jun signaling pathway mediates TMZ-upregulated miR-128 expression

**Fig 4:**
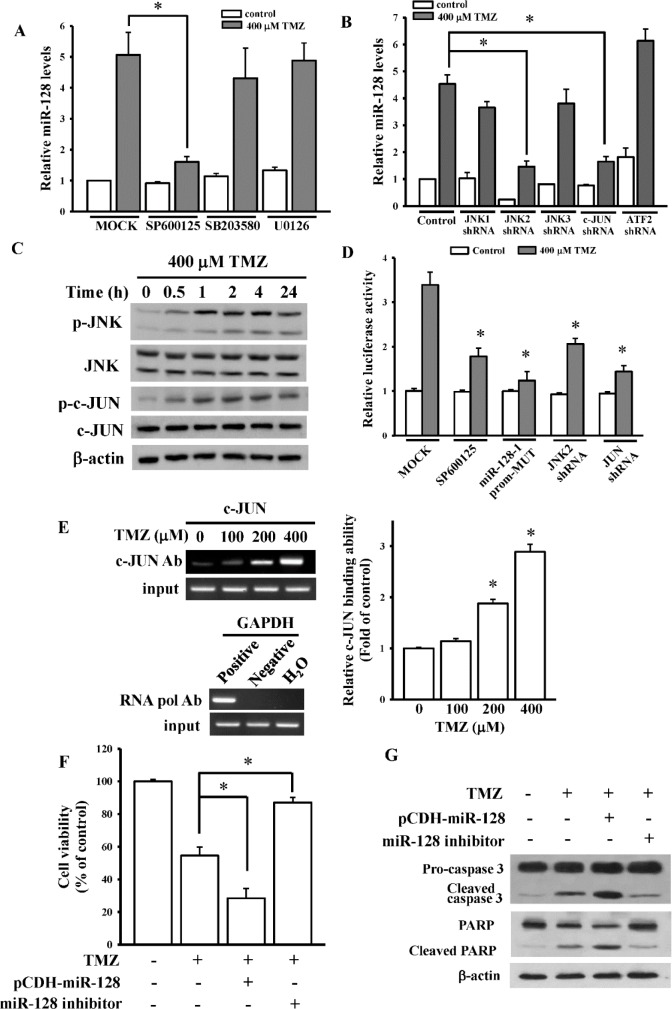
The JNK2/c-Jun signaling pathway is involved in temozolomide (TMZ)-upregulated miR-128 expression. (A) Effects of mitogen-activated protein kinase (MAPK) inhibitors including U0126 (an ERK inhibitor), SB203580 (a p38 inhibitor), and SP600125 (a JNK inhibitor) on endogenous miR-128 levels. (B) Inhibition of JNK2 and c-Jun expressions inhibited TMZ-upregulated miR-128 levels. After cells were respectively pretreated with 10 μM U0126, 5 μM SB203580, and 10 μM SP600125 for 1 h or respectively transfected with 1 μg of shRNAs for 24 h, 400 μM TMZ was added for another 24 h. miR-128 levels were measured through real-time PCR. Data are the mean ± SD of three experiments. * *p* < 0.05. (C) Time-dependent effects of TMZ on JNK and c-Jun activation. After cells were treated with 400 μM TMZ for the indicated time, phosphorylated and total forms of JNK and c-Jun were detected using immunoblotting assays. (D) SP600125 treatment, mutation of the activator protein (AP)-1-binding site, and inhibition of JNK2 or c-JUN attenuated TMZ-activated miR-128 promoter activity. After cells were transfected with 500 ng pGL3-miR-128-1-prom2500 or a pGL3-miR-128-1-prom2500 mutant for 24 h, luciferase activity was measured with SP600125 pretreatment for 1 h or cotransfected with 1 μg of shRNAs for 24 h followed by TMZ treatment for another 24 h. pNL1.1.TK[*Nluc*/TK] plasmids at 5 ng were also cotransfected into cells, and the NanoLucR luciferase value was used as an internal control. (E) The ChIP analysis showed that TMZ enhanced c-Jun binding to the miR-128 promoter. Procedures of the ChIP assay are described in “Materials and methods”. As positive or negative controls, protein–DNA complexes were incubated with anti-RNA polymerase or control mouse IgG antibodies. The input DNA represented one-fifth of the starting material. The right panel figure shows quantitative results from the left panel by densitometry. Overexpression and knockdown effects of miR-128 on TMZ-inhibited cell viability (F) and apoptosis-related protein expressions (G). After cells were respectively transfected with 750 ng of miR-128-overexpressing plasmids and 100 nM miR-128 inhibitor followed by TMZ treatment for another 24 h, cell viability and apoptosis-related protein expressions were measured using MTT and immunoblot assays. Data are the mean ± SD of three experiments. * *p* < 0.05.

A study suggested that the MAPK pathway is involved in TMZ-induced cell death [22]. Therefore, we investigated the effects of MAPK on TMZ-upregulated miR-128 expression. As presented in Fig 4A, only SP600125 (a JNK inhibitor), but not SB203580 (a p38 inhibitor) or U0126 (an ERK inhibitor), significantly reduced TMZ-mediated miR-128 levels. By performing shRNA transfections (S3A Fig), we observed that the inhibition of JNK2 and c-Jun, a major downstream regulator of JNK, suppressed TMZ-induced miR-128 levels (Fig 4B). In addition, we observed that TMZ enhanced both JNK and c-Jun phosphorylation in a time-dependent manner (Fig 4C). SP600125 treatment and knockdown of JNK2 or c-JUN significantly reduced TMZ-enhanced miR-128 promoter activity (Fig 4D). Furthermore, the JASPAR database [23] predicted one putative c-Jun-binding site in the core region (S4 Fig). After mutating seven nucleotides of the putative c-Jun-binding site in the miR-128 promoter, the mutation had the strongest inhibitory effect on miR-128 promoter activity with TMZ treatment (Fig 4D). The ChIP assay indicated that compared with the control without TMZ treatment, TMZ treatment induced the binding of more c-Jun proteins to the miR-128 promoter in a dose-dependent manner (Fig 4E). These findings support the involvement of JNK2/c-Jun in the TMZ-mediated miR-128 signaling pathway. In addition, overexpression and knockdown of miR-128 levels with pCDH-miR-128 and an miR-128 inhibitor significantly affected TMZ-mediated cell viability (Fig 4F), caspase 3 activation, and PARP degradation (Fig 4G), suggesting that miR-128-mediated signaling pathways are involved in TMZ cytotoxicity.

### Overexpression of miR-128 induces glioma cell death

**Fig 5:**
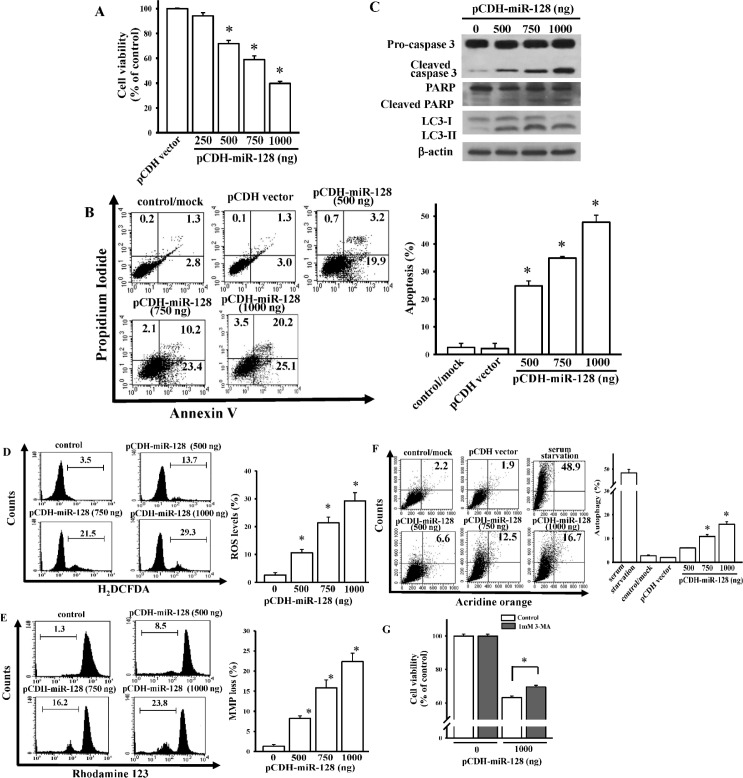
Identification of roles of miR-128 in mediating glioma cell death. Effects of miR-128 overexpression on cell viability (A), apoptosis ratio (B), apoptosis-related proteins levels (C), reactive oxygen species (ROS) generation (D), loss of the mitochondrial membrane potential (MMP) (E), and autophagy formation (F). After cells were transfected with the indicated dose of miR-128-expressing plasmids for 24 h, cell viability, caspase-3 activation, poly(ADP ribose) polymerase (PARP) degradation, LC3-I/II levels, apoptosis, ROS generation, loss of the MMP, and autophagy were measured using MTT assay, immunoblot assays, flow cytometry with annexin V/propidium iodide double-staining, H_2_DCFDA staining, rhodamine 123, and acridine orange staining, respectively. The right panel shows quantitative results from the left panel. Data are the mean ± SD of three independent experiments. * *p* < 0.05. (G) Effects of 3-MA on miR-128-induced autophagy. After cells were transfected with the indicated dose of miR-128-expressing plasmids for 4 h followed by 1 mM 3-MA treatment for another 24 h, cell viability was measured using the MTT assay. Data are the mean ± SD of three independent experiments. * *p* < 0.05.

We identified miR-128 putative target genes by using miRWalk 2.0 [24] and selected 3334 genes by at least three algorithm predictions. The ingenuity pathway analysis demonstrated that most of the miR-128 putative target genes participated in cell death, survival, and development (S2 Table), suggesting the involvement of miR-128 in cell fate decisions, particularly in cancers. To explore the role of miR-128 in regulating glioma cell death, we cloned *miR-128* into expressing plasmids, named pCDH-miR128. The overexpression of miR128 significantly enhanced intracellular endogenous levels of miR-128 (S3B Fig). We observed that increased miR-128 levels dose-dependently reduced cell viability (Fig 5A), enhanced the apoptosis ratio (Fig 5B), and affected apoptotic marker expressions (Fig 5C). Furthermore, we observed that miR-128 significantly promoted ROS generation (Fig 5D) and loss of the MMP (Fig 5E), suggesting that miR-128 induced a canonical apoptotic pathway in glioma cell death.

Because autophagy has been suggested as a new therapeutic mechanism in gliomas [25], we evaluated the effects of miR-128 on autophagy formation in U87MG glioma cells. As presented in Fig 5F, miR-128 resulted in a mild but statistically significant increase in autophagy generation (Fig 5F). 3-MA, an autophagy inhibitor, exerted inhibitory effects on miR-128-induced autophagy (S5 Fig) and mildly reversed miR-128-reduced cell viability (Fig 5G). Taken together, nonprotective autophagy is involved in miR-128-mediated glioma cell death.

### miR-128 directly targets and inhibits mTOR pathways

**Fig 6:**
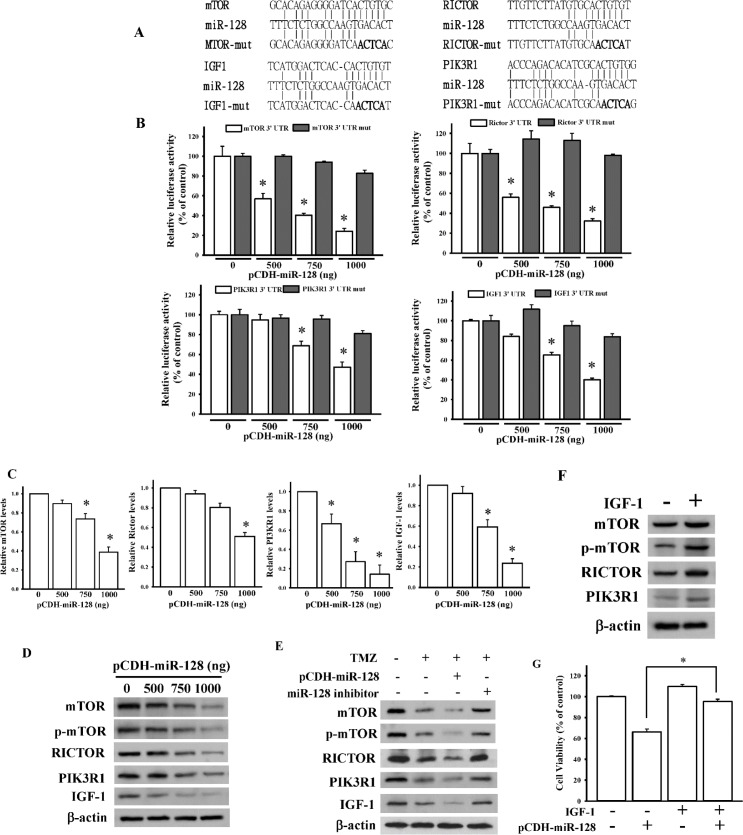
TMZ inhibited mammalian target of rapamycin (mTOR) pathways through miR-128 targeting. (A) Schematic diagram of potential miR-128-targeted sites in the 3’-untranslated region (UTR) of *mTOR*, *RICTOR*, *IGF1*, and *PIK3R1* genes. (B) Effects of miR-128 on 3’-UTR luciferase activity of *mTOR*, *RICTOR*, *IGF1*, and *PIK3R1* genes. To examine the effect of miR-128, different doses of miR-128-expressing plasmids were cotransfected with 500 ng pmiRGlo-3’-UTR or mutant 3’-UTR of *mTOR*, *RICTOR*, *IGF1*, and *PIK3R1* genes. Luciferase activity was measured in these cells 24 h after transfection. Effects of miR-128 overexpression on mRNA (C) and protein (D) expressions of *mTOR*, *RICTOR*, *IGF1*, and *PIK3R1* genes. After cells were respectively transfected with the indicated dose of miR-128-expressing plasmids for 24 h, relative mRNA and protein levels of the *mTOR*, *RICTOR*, *IGF1*, and *PIK3R1* genes were analyzed through real-time PCR and immunoblotting assays. Data are the mean ± SD of three experiments. * *p* < 0.05. (E) miR-128 is involved in TMZ-reduced mTOR signaling. (F) The effects of IGF-1 stimulation on miR-128-targeted genes expression levels. (G) IGF-1 stimulation attenuated miR-128-induced cytotoxicity. After cells were transfected with 750 ng of miR-128-expressing plasmids or 100 nM of a miR-128 inhibitor, 400 μM of TMZ was added for another 24 h. For IGF-1 stimulation assays, cells were treated with 200ng/mL of IGF-1 recombinant proteins for 24 h or transfected with 500 ng of miR-128-expressing plasmids combined with 200ng/mL of IGF-1 treatment for 24 h. Protein levels of mTOR, RICTOR, IGF1, and PIK3R1 were analyzed using immunoblotting assays. Cell viability was measured using the MTT assay. Data are the mean ± SD of three experiments. * *p* < 0.05.

mTOR signaling plays crucial roles in apoptosis and autophagy and is recognized as a targeted therapy for glioblastomas [16]. By using the TargetScan 6.2 prediction [19], we identified *IGF1*, *PIK3R1*, *PDK1*, *RICTOR*, *mTOR*, and *p70S6K1*, key components in canonical mTOR signaling, as putative target genes of miR-128 (Fig 6A and S6A Fig). Because p70S6K1 was identified as a target gene of miR-128 [11], we focused on the effects of miR-128 on expressions of other mTOR signaling-related proteins. To further confirm that *IGF1*, *PIK3R1*, *PDK1*, *RICTOR*, and *mTOR* are miR-128 target genes, 3’-UTRs of all these genes containing a miR-128-binding site were respectively cloned into the pmiRGlo-reporter plasmid to perform 3’UTR reporter assays. As presented in Fig 6B, different concentrations of miR-128-overexpressing plasmids significantly reduced luciferase activities of IGF1, PIK3R1, RICTOR, and mTOR. However, miR-128 had no effects on the luciferase activities of PDK1 (S6B Fig). To further validate that *IGF1*, *PIK3R1*, *RICTOR*, and *mTOR* are direct target genes of miR-128, five nucleotides located in the critical binding region of 3’-UTRs of the four genes were mutated through site-directed mutagenesis (Fig 6A). This procedure reduced or abolished the binding of miR-128 to these four genes. As presented in Fig 6B, miR-128 had no effect on luciferase activity after mutating the miR-128-targeted site. We also directly examined the effect of miR-128 on the expression of these four genes and observed that transient transfection of miR-128 into U87MG cells significantly and dose-dependently reduced mRNA and protein levels of IGF1, PIK3R1, RICTOR, and mTOR, as measured through real-time qPCR (Fig 6C) and immunoblotting analysis (Fig 6D). The overexpression of miR-128 had no effects on PDK1 mRNA and protein levels (S6D Fig). To further evaluate the involvement of miR-128 in TMZ-repressed mTOR signaling, we measured mTOR signaling-related protein levels after transfection of miR-128-overexpressing plasmids or an inhibitor combined with TMZ treatment. As shown in Fig 6E, overexpression or knockdown of miR-128 expression significantly influenced TMZ-reduced mTOR signaling. We examined the effects of mTOR signaling-related proteins on miR-128 cytotoxicity and observed that IGF-1 stimulation enhanced the endogenous levels of mTOR, PIK3R1, and RICTOR (Fig 6F). Furthermore, IGF-1 stimulation significantly reduced miR-128-reduced cell viability (Fig 6G). Taken together, TMZ-mediated miR-128 upregulation inhibited mTOR signaling expressions, resulting in U87MG cell death.

## Discussion

In this study, we explored TMZ-mediated miRNA gene profiles of U87MG glioma cells containing five significantly upregulated and 17 significantly downregulated miRNAs. miR-128 was one of the TMZ-upregulated miRNAs. The expression level of miR-128 was lower than that of normal astrocytes in glioma cell lines, and low miR-128 expression levels were strongly associated with poor survival. TMZ dose-dependently enhanced miR-128 expression levels and promoter activities through regulating *miR-128-1* but not *miR-128-2*. Furthermore, JNK2/c-Jun pathways were identified to be involved in TMZ-induced miR-128 expression through transcriptional regulation. In addition, overexpression or knockdown of miR-128 expression affected TMZ cytotoxicity. The overexpression of miR-128 alone significantly enhanced apoptotic cell death, ROS generation, MMP loss, and nonprotective autophagy formation. The key members of canonical mTOR pathways, including *mTOR*, *RICTOR*, *IGF1*, and *PIK3R1*, were identified as direct target genes of miR-128. The overexpression or knockdown of miR-128 expression significantly affected TMZ-repressed mTOR pathways. All these results indicate that TMZ can induce apoptotic cell death of glioblastoma cells through upregulating miR-128 gene expression and that miR-128-inhibited mTOR cascades are involved in TMZ cytotoxicity.

The prodrug TMZ, an imidazole derivative, was developed as a second-generation oral alkylating chemotherapeutic agent for aggressive pituitary adenomas and GBM. Through methylation of the O(6) position of guanine, TMZ induces DNA damage resulting in cytotoxic DNA adduct accumulation and cell apoptosis accompanied by mitochondrial dysfunction, endoplasmic reticular stress, and autophagy formation [26]. Several signaling cascade mediators have been identified to be involved in TMZ cytotoxicity, such as the Mcl-1/Bak axis [27], heat-shock proteins (MSH2, HSPA, and HSPB1) [28], and p53 [29]. However, no studies have comprehensively analyzed TMZ-mediated gene networks or miRNA signatures. In this study, we conducted an miRNA microarray analysis and observed the DE of 248 miRNAs in TMZ-treated U87MG cells, including upregulation of miR-128. TMZ treatment significantly enhanced intracellular miR-128 levels. In addition, we observed the involvement of miR-128 in TMZ-induced apoptotic cell death of glioma cells. Increasing number of studies have reported that miRNAs can enhance the sensitivity of glioma cells to TMZ or improve TMZ-induced apoptosis of miR-181b [30], and miR-143 [31]. Furthermore, antisense miRNA has been used as an adjuvant agent for evaluating the effects of TMZ on glioma cells [32, 33]. Taken together, elucidation of TMZ-mediated miRNA networks can provide novel directions for understanding TMZ-mediated cytotoxic mechanisms.

Because miR-128 participates in tumorigenesis and metastasis [8], investigation of transcriptional mechanisms directly regulating miR-128 gene expression is critical for carcinogenesis. Several transcription factors have been identified to mediate miR-128 gene levels. Snail family zinc finger (SNAIL), an epithelial–mesenchymal transition regulator, directly downregulated miR-128-2 expression through TGF-β signaling [34]. By contrast, p53 can bind to the miR-128-1 promoter and activate miR-128 expression levels [35]. The p53R175H protein, a hotspot p53 mutant, transactivates miR-128-2 promoter activities, followed by miR-128 upregulation [36]. In addition, increasing evidence has suggested that members of the MAPK family, including JNK, p38, and ERK, are crucial downstream regulators of TMZ cytotoxicity [37–39]. By using a promoter reporter assay and ChIP analysis, we validated that JNK2/c-Jun pathways can bind to the miR-128-1 gene promoter and promote miR-128 expression upon TMZ treatment. All these findings suggest that TMZ-activated JNK2/c-Jun pathways can be another mechanism regulating miR-128 gene expression.

Several miR-128 target genes have gradually been identified including the p70S6K1 protein [11]. P70S6K1, one of the key downstream targets of mTOR, is involved in tumorigenesis and chemotherapeutic drug resistance. The overexpression of miR-128 significantly repressed p70S6K1 protein levels and its downstream hypoxia-inducible factor (HIF)-1 and vascular endothelial growth factor (VEGF) signaling. Moreover, p70S6K1 and members of mTOR signaling, including mTOR, RICTOR, IGF1, PIK3R1, and PDK1, have been predicted to be direct target genes of miR-128. By using the 3’-UTR reporter assay, real-time qPCR, and immunoblotting assays, we observed that miR-128 directly targets and inhibits mTOR, RICTOR, IGF1, and PIK3R1 but not PDK1 expressions. Similar to findings in a previous study [40], our study results revealed that TMZ treatment alone significantly inhibited mTOR signaling. Furthermore, we observed that the overexpression or knockdown of miR-128 expression affected TMZ-repressed mTOR signaling, suggesting that miR-128-targeted mTOR signaling is involved in TMZ-mediated glioma cell death.

In summary, we observed that TMZ treatment significantly affected intracellular miRNA expression in glioma cells. Furthermore, we observed that TMZ upregulated miR-128 levels through JNK2/c-Jun transcriptional activation of the miR-128-1 gene. Finally, we determined that miR-128 directly targets mTOR signaling and is involved in TMZ cytotoxicity. In addition to miR-128, TMZ-treated microarray results revealed an increase in the miR-320b level and a decrease in miR-765 and miR-762 levels (Table 1). A study suggested that miR-320b suppressed colorectal cancer cell proliferation by targeting c-Myc [41]. By contrast, miR-765 and miR-762 promoted cell proliferation in hepatocellular carcinoma [42] and breast cancer [43], respectively. However, no studies have reported their roles in gliomagenesis, which should be investigated in the future. Furthermore, our study has some limitations. The JNK2/c-Jun signaling-mediated miR-128 expression is one of the TMZ-regulated downstream pathways in GBM. Most of the TMZ-mediated mRNA/miRNA networks remain unclear. Because TMZ is a DNA-damaging drug, downstream pathways including miR-128 signaling are secondary effects following the DNA damage response. Thus, connecting mechanisms between the DNA damage response and TMZ-regulated downstream pathways is crucial. The *in vivo* effects of miR-128 combined with TMZ treatment should be investigated in the future. Our findings suggest the involvement of miR-128-inhibited mTOR pathways in TMZ-mediated cytotoxicity and provide novel mechanisms for investigating GBM development.

## Supporting Information

S1 Fig Cytotoxicity effects of temozolomide (TMZ) on U87-MG cell viability. After cells were treated with indicated doses of TMZ for 24 h, cell viability was measured with an MTT assay. Data are the mean ± SD of three experiments. * *p* < 0.05.
(TIFF)

S2 Fig Comparison of endogenous levels and promoter activity between miR-128-1 and miR-128-2. (A) Endogenous expression levels of primary miR-128-1 and miR-128-2. Relative expression levels were measured using a real-time PCR. (B) Relative promoter activities of the *miR-128-1* and *miR-128-2* genes. After cells were respectively transfected with 500 ng pGL3-miR-128-1-prom and pGL3-miR-128-2-prom for 24 h, luciferase activity was measured. pNL1.1.TK[*Nluc*/TK] plasmids at 5 ng were also co-transfected into cells, and the NanoLucR luciferase value was used as an internal control. Data are the mean ± SD of three experiments.
(TIFF)

S3 Fig Effects of shRNA and miR-128-overexpressing plasmids on indicated gene expressions and endogenous miR-128 levels. (A) The knock-down effects on *JNK1*, *JNK2*, *JNK3*, *c-Jun*, and *ATF2* gene expressions by shRNA transfection. After cells were respectively transfected with 1 μg shRNA for 24 h, cells were collected to measure protein expressions with an immunoblotting assay. (B) Measurement of endogenous miR-128 levels after miR-128 overexpression. After respectively transfecting miR-128-overexpressing plasmids at the indicated dose for 24 h, cells were collected to measure the relative expression levels of miR-128 using a real-time PCR. Data are the mean ± SD of three experiments.
(TIFF)

S4 Fig Prediction of the putative activator protein (AP)-1-binding site on the *miR-128-1* promoter in the -1970 to -2050 bp region. (A) Schematic diagram shows the putative AP-1-binding sequence. (B) The putative AP-1-binding site (red color) was predicted by the JASPAR database.
(TIFF)

S5 Fig Effects of 3-MA on cell viability and autophagy formation. (A) The cytotoxicity of 3-MA against U87-MG cell viability. After cells were treated with indicated doses of 3-MA for 24 h, cell viability was measured by an MTT assay. (B) 3-MA reduced miR-128-enhanced autophagy generation. After transfection with 750 ng of miR-128-overexpressing plasmids for 4 h followed by 24 h of treatment with 1 mM 3-MA, cells were collected. The autophagy percentage was measured by flow cytometry with acridine orange staining. Data are the mean ± SD of three experiments. * *p* < 0.05.
(TIFF)

S6 Fig Identification that PDK1 is not a direct target gene of miR-128. (A) Schematic diagram of potential miR-128-targeted sites in the PDK1 3’-untranslated region (UTR). (B) Effects of miR-128 on PDK1 3’-UTR luciferase activity. To test for miR-128's effect, different doses of miR-128 plasmids were co-transfected with 500 ng of the pmiRGlo-PDK1 3’-UTR. Luciferase activity was measured in these cells 24 h after transfection. Effects of miR-128 overexpression on PDK1 mRNA (C) and protein (D) expressions. After cells were respectively transfected with the indicated dose of miR-128 plasmids for 24 h, the relative mRNA and protein levels of PDK1 were analyzed using a real-time PCR and immunoblotting assay.
(TIFF)

S1 File Array data of TMZ-mediated microRNA expressions. (XLSX)

S1 Table Primer list. (DOCX)

S2 Table Prediction of top 10 miR-128-regulated signaling pathways. (DOCX)
