# Supplementary figures and images for: The Inhibition of microRNA-128 on IGF-1-Activating mTOR Signaling Involves in Temozolomide-Induced Glioma Cell Apoptotic Death
Source: PLoS One. 2016 Nov 28;11(11):e0167096. doi: 10.1371/journal.pone.0167096 (PMC5125683; doi:10.1371/journal.pone.0167096)

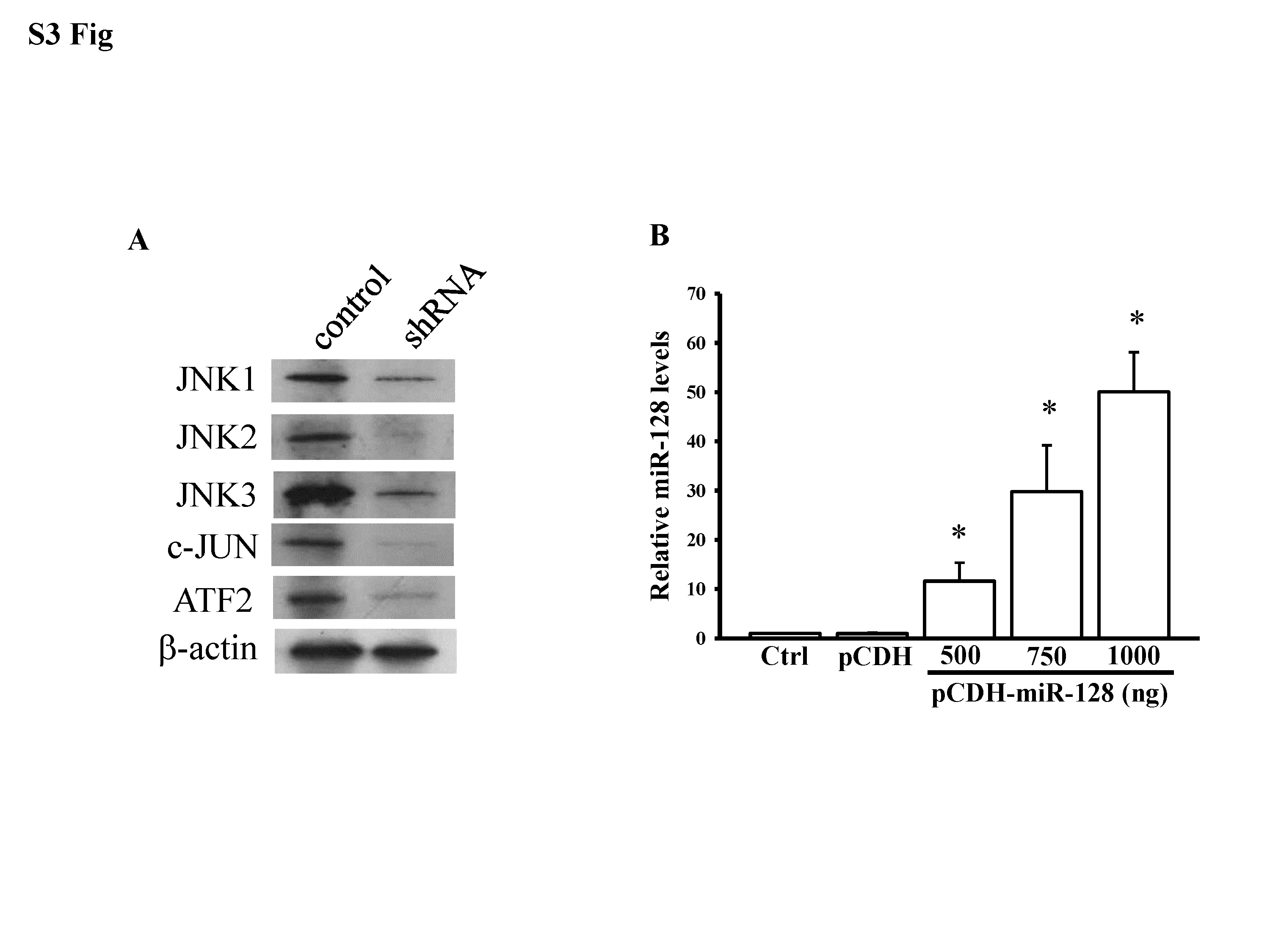

Supplement: S3 Fig — (A) The knock-down effects on JNK1, JNK2, JNK3, c-Jun, and ATF2 gene expressions by shRNA transfection. After cells were respectively transfected with 1 μg shRNA for 24 h, cells were collected to measure protein expressions with an immunoblotting assay. (B) Measurement of endogenous miR-128 levels after miR-128 overexpression. After respectively transfecting miR-128-overexpressing plasmids at the indicated dose for 24 h, cells were collected to measure the relative expression levels of miR-128 using a real-time PCR. Data are the mean ± SD of three experiments. (TIFF) [file pone.0167096.s003.tiff]

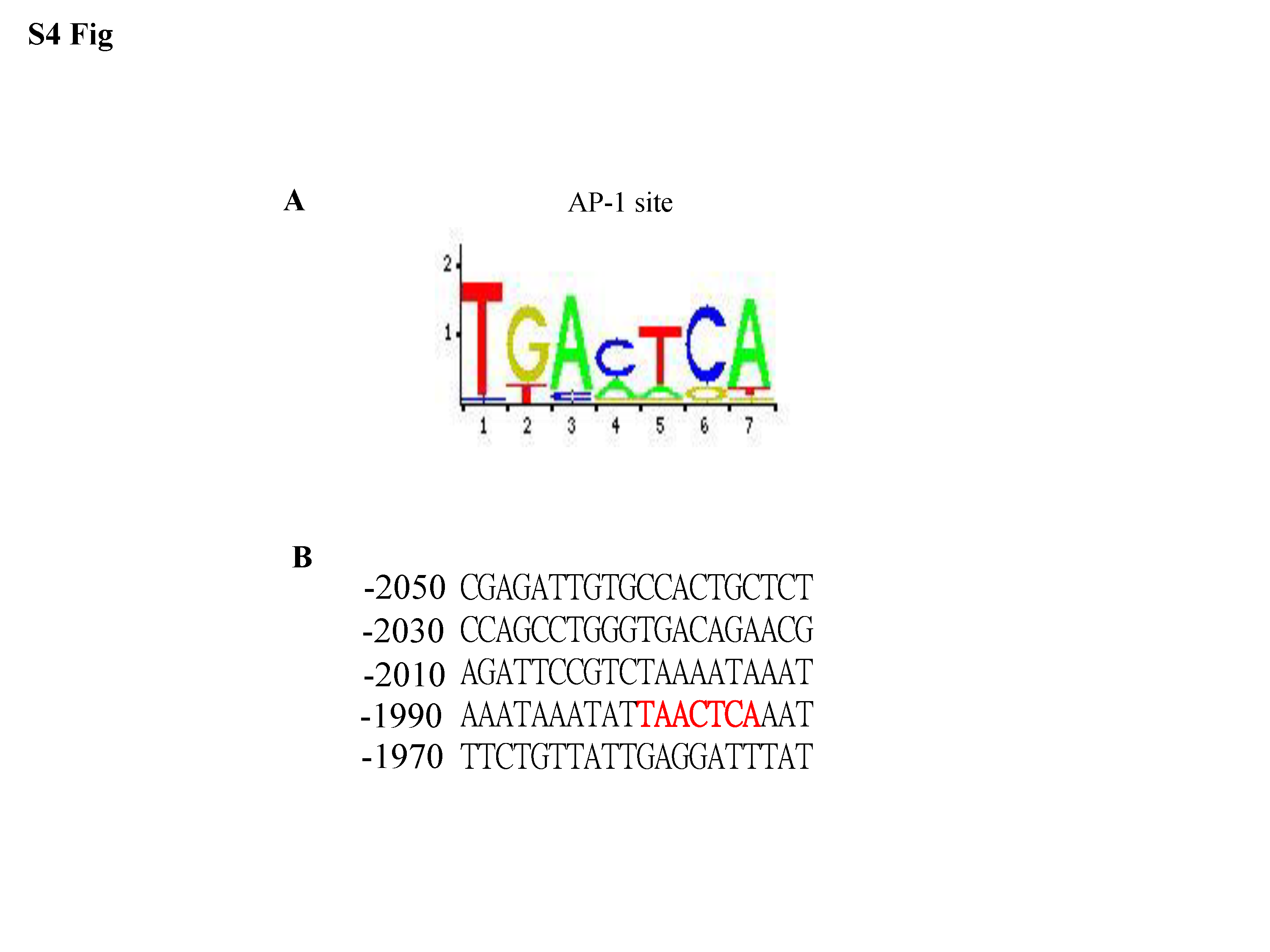

Supplement: S4 Fig — (A) Schematic diagram shows the putative AP-1-binding sequence. (B) The putative AP-1-binding site (red color) was predicted by the JASPAR database. (TIFF) [file pone.0167096.s004.tiff]

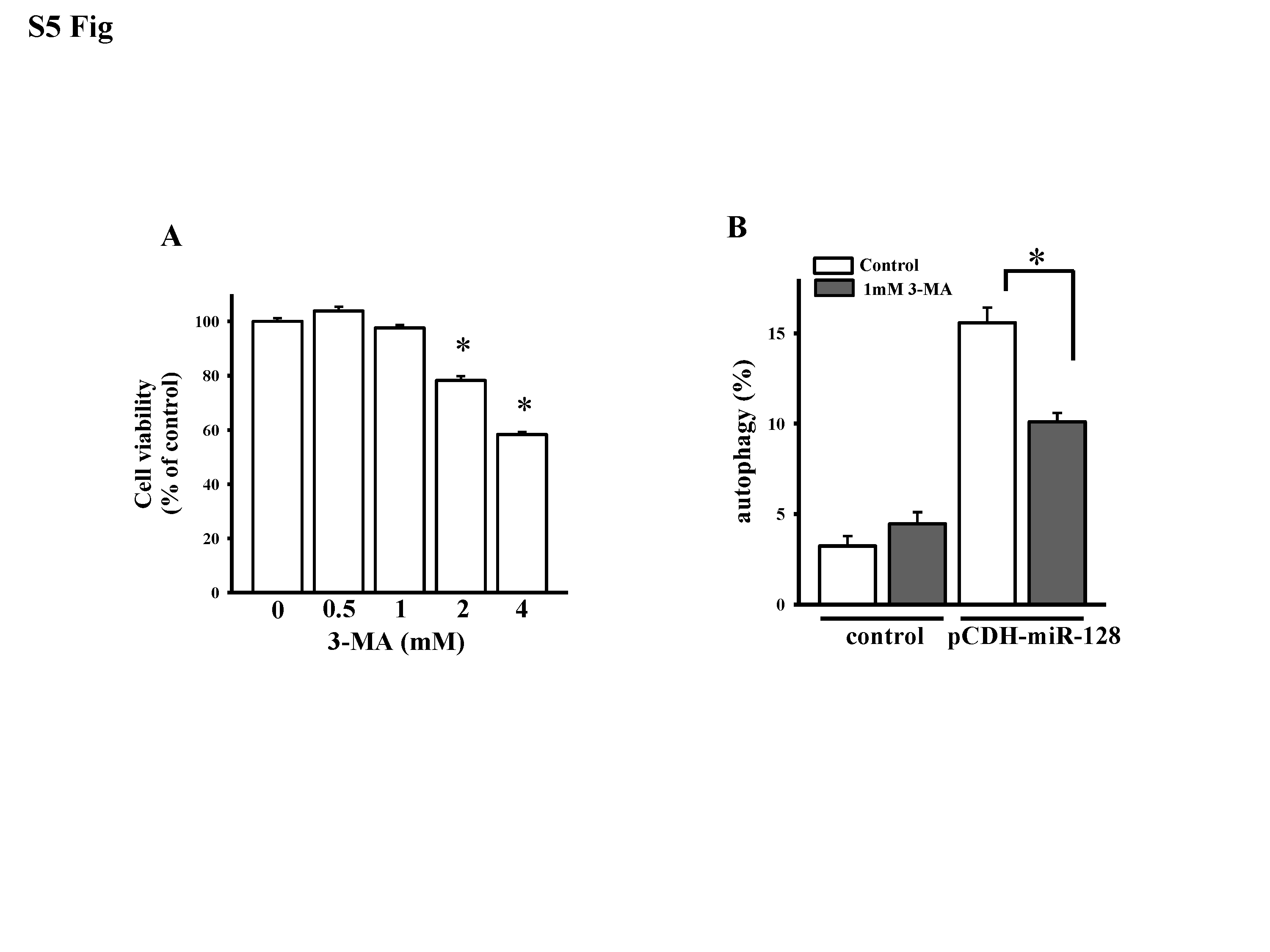

Supplement: S5 Fig — (A) The cytotoxicity of 3-MA against U87-MG cell viability. After cells were treated with indicated doses of 3-MA for 24 h, cell viability was measured by an MTT assay. (B) 3-MA reduced miR-128-enhanced autophagy generation. After transfection with 750 ng of miR-128-overexpressing plasmids for 4 h followed by 24 h of treatment with 1 mM 3-MA, cells were collected. The autophagy percentage was measured by flow cytometry with acridine orange staining. Data are the mean ± SD of three experiments. * p < 0.05. (TIFF) [file pone.0167096.s005.tiff]

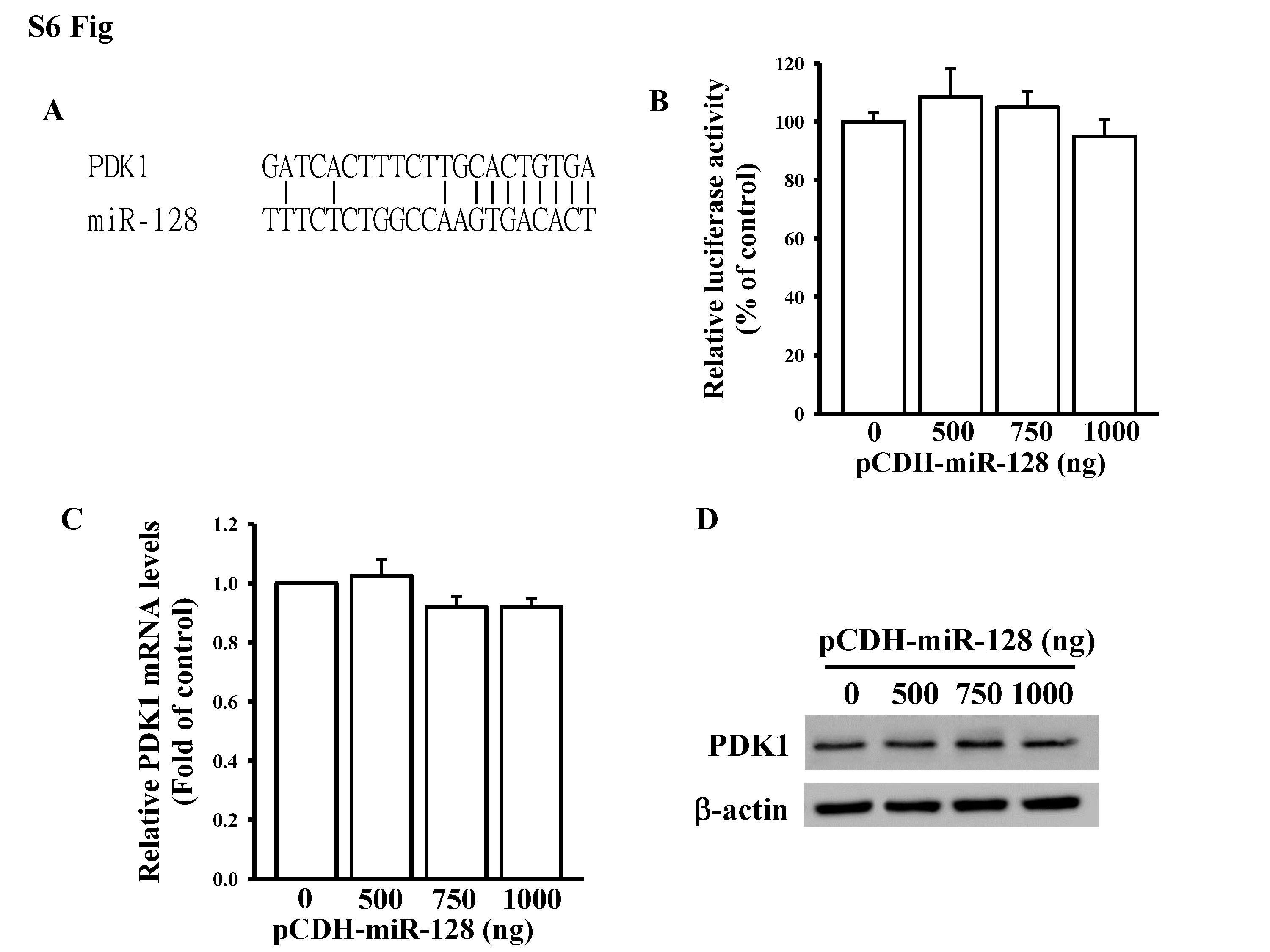

Supplement: S6 Fig — (A) Schematic diagram of potential miR-128-targeted sites in the PDK1 3’-untranslated region (UTR). (B) Effects of miR-128 on PDK1 3’-UTR luciferase activity. To test for miR-128's effect, different doses of miR-128 plasmids were co-transfected with 500 ng of the pmiRGlo-PDK1 3’-UTR. Luciferase activity was measured in these cells 24 h after transfection. Effects of miR-128 overexpression on PDK1 mRNA (C) and protein (D) expressions. After cells were respectively transfected with the indicated dose of miR-128 plasmids for 24 h, the relative mRNA and protein levels of PDK1 were analyzed using a real-time PCR and immunoblotting assay. (TIFF) [file pone.0167096.s006.tiff]
