# Supplementary material for: The Inhibition of microRNA-128 on IGF-1-Activating mTOR Signaling Involves in Temozolomide-Induced Glioma Cell Apoptotic Death
Source: PLoS One. 2016 Nov 28;11(11):e0167096. doi: 10.1371/journal.pone.0167096 (PMC5125683; doi:10.1371/journal.pone.0167096)
Supplement: S1 Table — (DOCX) [file pone.0167096.s008.docx]

**S1 Table. Primer list**

| Primer name | Sequence |
| --- | --- |
| For promoter cloning | |
| miR-128-1 prom2500-F | ATAACGCGTCTGATGTTAGTCCCATAGTT |
| miR-128-1 prom2500-R | ATACTCGAGGCACTTGCCGAGGAGGACTCA |
| miR-128-1 promoter1500-F | ATAACGCGTGCTTGTCCATCTGTTTCATAA |
| miR-128-1 promoter1000-F | ATAACGCGTATTTCTGGTTGGTGTACAGATC |
| miR-128-1 promoter500-F | ATAACGCGTCAGATTAAGAAATAAGGAATTGT |
| miR-128-2 promoter-F | ATAACGCGTGCTGAATGCTAATTATCTACAT |
| miR-128-2 promoter-R | ATACTCGAGTGAGCCACGCGCAGCAGCGTGA |
| For promoter mutagenesis | |
| miR-128-1 prom-Mut-F | CACTGCTCTCCAGCCTGGGACGAGATTCCGTCTAAAAT |
| miR-128-1 prom-Mut-R | ATTTTAGACGGAATCTCGTCCCAGGCTGGAGAGCAGTG |
| For ChIP assay | |
| miR-128-ChIP-F | CCTGGGTGACAGAACGAGAT |
| miR-128-ChIP-R | AACATGCTACCGAGGTCAGG |
| For miR-128-1 gene cloning | |
| MIR128-1-clone-F | ATAGAATTCTTGAATACTGTGAAGTACACTGC |
| MIR128-1-clone-R | CCGGGATCCTAAGCAATAGCTTTCACAAATT |
| For real-time PCR | |
| mTOR sybr-F | ATGCTTGGAACCGGACCTG |
| mTOR sybr-R | TCTTGACTCATCTCTCGGAGTT |
| IGF1-syber-F | GCTCTTCAGTTCGTGTGTGGA |
| IGF1-syber-R | GCCTCCTTAGATCACAGCTCC |
| PDK1-syber-F | CTGTGATACGGATCAGAAACCG |
| PDK1-syber-R | TCCACCAAACAATAAAGAGTGCT |
| PIK3R1-syber-F | AAGAAGTTGAACGAGTGGTTGG |
| PIK3R1-syber-R | GCCCTGTTTACTGCTCTCCC |
| RICTOR-syber-F | GCTAGGTGCATTGACATACAACA |
| RICTOR-syber-R | AGTGCTAGTTCACAGATAATGGC |
| 18S-rRNA-sybr-F | GTAACCCGTTGAACCCCATT |
| 18S-rRNA-sybr-R | CCATCCAATCGGTAGTAGCG |
| For 3’ UTR cloning |  |
| IGF1-3UTR-F | TAAGAGCTCGAAAGAGTCTGGCCAAAACG |
| IGF1-3UTR-R | TAACTCGAGAGAATTCCTTGCATCTCAGCA |
| MTOR-3UTR-3F | TAAGAGCTCCTGGAGGCCCAGATGTGCCCATCACGTTT |
| MTOR -3UTR-3R | TAACTCGAGTACATATGTTTAAAATTCTGATGTCATTTA  |
| PDK1-3UTR-F | TAAGAGCTCTTCTGGGAGAGCTTTTTCCT |
| PDK1-3UTR-R | TAACTCGAGGTGCAAGCCCCCTAATTACC |
| PIK3R1-3UTR-F | TAAGAGCTCGGGAAGACATGCAGCCTAAG |
| PIK3R1-3UTR-R | TAACTCGAGTTTTCTGTTGCTGGGTTTTTG |
| RICTOR-3UTR-3F | TAAGAGCTCCCAGCTGGACTGATTAGGATTC |
| RICTOR -3UTR-3R | TAACTCGAGCCAAGACCAAAGACCACGAT |
| For 3’ UTR mutagenesis |  |
| IGF1-3UTR-MUT-F | GTGTCATGGACTCACCAACTCATGACCTTGGGCAAGTCA |
| IGF1-3UTR-MUT-R | TGACTTGCCCAAGGTCATGAGTTGGTGAGTCCATGACAC |
| MTOR-3UTR-MUT-F | ATGCACAGAGGGGATCAACTCACAGTGGGACCACCCTCA |
| MTOR-3UTR-MUT-R | TGAGGGTGGTCCCACTGTGAGTTGATCCCCTCTGTGCAT |
| PIK3R1-3UTR-MUT-F | GACCCAGACACATCGCAACTCAGATTATTTCATTTTGTA |
| PIK3R1-3UTR-MUT-R | TACAAAATGAAATAATCTGAGTTGCGATGTGTCTGGGTC |
| RICTOR-3UTR-MUT-F | AATTGTTCTTATGTGCAACTCATAGCAACTTACATTAT |
| RICTOR-3UTR-MUT-R | ATAATGTAAGTTGCTATGAGTTGCACATAAGAACAATT |
