# Supplementary material for: The Inhibition of microRNA-128 on IGF-1-Activating mTOR Signaling Involves in Temozolomide-Induced Glioma Cell Apoptotic Death
Source: PLoS One. 2016 Nov 28;11(11):e0167096. doi: 10.1371/journal.pone.0167096 (PMC5125683; doi:10.1371/journal.pone.0167096)
Supplement: S2 Table — (DOCX) [file pone.0167096.s009.docx]

**S2 Table. Prediction of top 10 miR-128-regulated signaling pathways**

| **Score** | **Top Diseases and Functions** | **Focus Molecules** | **Molecules in Network** |
| --- | --- | --- | --- |
| 8 | Cell Death and Survival, Neurological Disease, Cell Cycle | 24 | B4GALT5, BCL2L11, CDC6, CDC25A, CDKN1B, CDKN1C, DLG4, DRD2, E2F2, E2F3, E2F6, EGFR, ETV4, GRIN1, GRIN2A, HTT, KLF4, MECP2, MET, MYBL2, NOS1, NUMB, PPARGC1A, PROM1, PTGS2, RB1, RBL1, RRM2, SP1, SP3, SYNM, TAF4, TFPI2, TRAF6, VKORC1L1 |
| 7 | Cellular Movement, Cancer, Organismal Injury and Abnormalities | 14 | CCSAP, FOXM1, GALNT2, GLI1, GREM2, GRIA3, HNRNPU, IPO7, NAV3, NR2F2, NREP, PGM2L1, PLXDC1, PTCH1, RAP2A, VEGFC |
| 6 | Metabolic Disease, Neurological Disease, Psychological Disorders | 20 | ABCA1, ACSL5, APOA1, APP, BACE1, DNM1, GALNT13, GFAP, GFPT2, GRB2, IGF1, IL1B, KIAA1324L, MAP2, MBP, NAT8B, NFIC, NFIX, NOS2, PEG10, PHLDA2, PRNP, SERPINA3, SHC1, SHC3, SORL1, SORT1, TAP2, VPS35 |
| 6 | Cell Death and Survival, Embryonic Development, Cancer | 20 | AKT1, APAF1, ATG5, ATG7, BAK1, BAX, BCL2L2, BID, BIRC2, CASP3, CASP7, CXCL8, CXCL12, CXCR4, DHCR24, FAS, FASLG, GSK3B, IGF1R, MAPK1, POU5F1, PSEN1, RIPK1, TNF, TNFRSF9, TNFRSF25, TNFRSF10B, TNFSF10, TRAF2, TRAF3, WEE1 |
| 5 | Gene Expression, Cell-To-Cell Signaling and Interaction, Neurological Disease | 18 | APBB1, APP, CLEC2D, CREB1, CREBBP, DUSP5, FOSB, IGFBP3, KLC1, LRP1, MCL1, miR-17, NR4A1, PARK2, PLK2, PRKCA, PVR, PXN, RGS2, SERPINE1, SMAD2, SNCA, SREBF2, SRF, TGFB1, TOR1A, UBE2L3, UBE3A |
| 5 | Cellular Movement, Cancer, Neurological Disease | 17 | ARHGDIA, BMI1, CADM1, CDC42, DNM2, ELMO1, ELOVL6, EMP2, EPHA4, FGF2, FZD4, IGFBP2, ITGB8, MDGA1, mir-218, NUP210, PDGFRA, PPARG, PTGFRN, PTPN11, RAC1, RBM4, RBMS3, SASH1, SRC, VAV3 |
| 3 | Cell Cycle, Organismal Development, Embryonic Development | 13 | CCND1, CDK4, CDK6, CDKN2A, CDKN2B, FUT4, ITGA3, ITGA5, ITGB1, ITGB3, JAG1, MBD1, MMP2, MMP14, NOTCH1, PAX8, PPIF, PROM1, SEL1L, STAT3, TERT, TIMP2 |
| 3 | Cell Death and Survival, Cancer, Organismal Injury and Abnormalities | 14 | AKT1, BAD, CASP8, CASP10, CBL, CFLAR, EGLN1, FADD, FAS, GTF3C1, HIF1A, LLGL1, MTOR, MYH10, MYO1D, NFKB1, PDPK1, PEA15, PIK3R1, POU5F1, PRKCI, PTEN, PTK2, RICTOR, RPS6KB1, RPTOR |
| 2 | Cardiovascular System Development and Function, Organismal Development, Cellular Development | 14 | ACHE, AKT2, BAD, BCL2L1, CASP8, CCNE1, CREBBP, CTSB, ELAVL1, FGF1, FOXM1, GNB2L1, IL6, IL6R, MMP2, MTDH, NF1, NFKBIA, NTN1, PLAUR, PRKCE, RELA, SLC1A2, SMAD4, STAT3, TFAP2A, TNF, TNFSF10, TRPV2, TWIST1, VCAM1, VEGFA, YY1 |
| 2 | Hematological System Development and Function, Tissue Morphology, Cell Death and Survival | 14 | BCL6, CCL2, CCL3, CCL4, CCNG2, CD40LG, CXCR4, DDIT3, FBXO32, FOXO1, FOXO3, GADD45B, HOXA9, HSPB2, ICAM1, IL1A, IL1B, KLF6, KLF10, MAP2K4, MAP3K11, MAPK8, MAPK9, MAX, MITF, MNT, MYC, POU2F1, SIN3A, SIN3B, SPP1, TNF, TXNIP, USF1, VCAM1 |
